# Mathematical model for bone mineralization

**DOI:** 10.3389/fcell.2015.00051

**Published:** 2015-08-21

**Authors:** Svetlana V. Komarova, Lee Safranek, Jay Gopalakrishnan, Miao-jung Yvonne Ou, Marc D. McKee, Monzur Murshed, Frank Rauch, Erica Zuhr

**Affiliations:** ^1^Faculty of Dentistry, McGill UniversityMontreal, QC, Canada; ^2^Shriners Hospital for Children-CanadaMontreal, QC, Canada; ^3^Department of Mathematics, Simon Fraser UniversityBurnaby, BC, Canada; ^4^The Fariborz Maseeh Department of Mathematics and Statistics, Portland State UniversityPortland, OR, USA; ^5^Department of Mathematical Sciences, University of DelawareNewark, DE, USA; ^6^Department of Anatomy and Cell Biology, Faculty of Medicine, McGill UniversityMontreal, QC, Canada; ^7^Department of Medicine, Faculty of Medicine, McGill UniversityMontreal, QC, Canada; ^8^Department of Mathematics, High Point UniversityHigh Point, NC, USA

**Keywords:** bone histomorphometry, matrix mineralization, mineralization inhibitors, nucleating centers, osteogenesis imperfecta, osteomalacia, X-linked hypophosphatemia, rickets

## Abstract

Defective bone mineralization has serious clinical manifestations, including deformities and fractures, but the regulation of this extracellular process is not fully understood. We have developed a mathematical model consisting of ordinary differential equations that describe collagen maturation, production and degradation of inhibitors, and mineral nucleation and growth. We examined the roles of individual processes in generating normal and abnormal mineralization patterns characterized using two outcome measures: mineralization lag time and degree of mineralization. Model parameters describing the formation of hydroxyapatite mineral on the nucleating centers most potently affected the degree of mineralization, while the parameters describing inhibitor homeostasis most effectively changed the mineralization lag time. Of interest, a parameter describing the rate of matrix maturation emerged as being capable of counter-intuitively increasing both the mineralization lag time and the degree of mineralization. We validated the accuracy of model predictions using known diseases of bone mineralization such as osteogenesis imperfecta and X-linked hypophosphatemia. The model successfully describes the highly nonlinear mineralization dynamics, which includes an initial lag phase when osteoid is present but no mineralization is evident, then fast primary mineralization, followed by secondary mineralization characterized by a continuous slow increase in bone mineral content. The developed model can potentially predict the function for a mutated protein based on the histology of pathologic bone samples from mineralization disorders of unknown etiology.

## Background

Defects in bone mineralization can result in reduced or excessive bone mineralization, which can lead to serious clinical manifestations, including bone deformities and fractures. Plasma levels of calcium and phosphate—ionic mineral constituents of bone hydroxyapatite mineral—as well as their key regulators parathyroid hormone, vitamin D and FGF23, are critically important for successful mineralization (Shimada et al., 2004; Morris et al., 2012). Numerous conditions which are not associated with abnormal levels of circulating calcium and phosphate are also known to result in hypo- or hypermineralization of bone matrix (Roughley et al., 2003). One example is osteogenesis imperfecta, a disease usually caused by mutations in collagen type I-encoding genes and characterized by increased bone mineralization (Roschger et al., 2008a; Forlino et al., 2011). The clinical phenotype of osteogenesis imperfecta can also be caused by mutations in genes encoding the proteins that are involved in collagen post-translational modifications such as bone morphogenetic protein 1 (BMP1), or the proteins that regulate bone mineralization by an as yet unknown mechanism (Marini et al., 2014). The mechanisms underlying the development of hypo- and hypermineralization of extracellular bone matrix when the plasma levels of calcium and phosphate are within the normal range are complex and not well understood.

Clinically, the mineralization process can be examined in bone biopsy samples, which are typically obtained from the iliac bone. When tetracycline labeling is performed prior to biopsy, it is possible to assess the mineralization process quantitatively using bone histomorphometry (Rauch, 2006). Key, well-accepted histomorphometric descriptors of the mineralization process include the average thickness of the layer of unmineralized organic bone matrix (osteoid thickness) and the duration of the lag time between the deposition of organic matrix and the start of mineralization (mineralization lag time). Using quantitative backscattered-electron imaging, it is also possible to determine the average density of mineralized bone (Roschger et al., 2008b), which among other measures indicates the proportion of the mineralized tissue mass contributed to by the mineral ions.

The molecular origin and mechanistic basis of bone hypo- and hypermineralization are incompletely understood; however, it is clear that the process of mineralization is tightly regulated and is highly nonlinear. The goal of this study was to develop a simplified mathematical model of a complex process that provides a description of basic steps in the mineralization process, including collagen production and maturation, delivery and degradation of inhibitors, as well as mineral nucleation and growth. To our knowledge, no mathematical model focused on bone mineralization process exists to date, however important work has been performed in modeling the role of mineralization process in determining the bone mineral distribution (Ruffoni et al., 2007); the process of dentinogenesis including dentin phosphoprotein-regulated mineralization (Niño-Barrera et al., 2013) as well as describing the mineralization-induced changes in mechanical properties of collagen (Crolet et al., 2005; Nikolov and Raabe, 2008; Barkaoui and Hambli, 2014). Using the intentionally simplified model which includes multiple complexities in a limited number of variables, we were able to capture the significant nonlinearity of the mineralization process. Modeling predictions regarding the roles of individual processes in generating abnormal mineralization patterns were compared to the phenotype of diseases having in major bone mineralization defects—namely osteomalacia diseases and osteogenesis imperfecta.

## Model development

We modeled the changes over time in the concentration of five key players in the mineralization process—the collagen matrix subdivided into naïve and mature matrix, the inhibitors of mineralization, the nucleation centers (nucleators), and the hydroxyapatite mineral—within a homogeneous unit volume of osteoid of ~1 μm^3^ in dimensions using a system of ordinary differential equations. The following considerations were used to identify the main model assumptions.

1. Physiologically, the formation of bone tissue begins with the secretion of an organic bone matrix by osteoblasts, which to a large extent (by weight and volume) consists of collagen type I (Christiansen et al., 2000). Once this naïve organic matrix is deposited into the extracellular compartment, it needs to be processed in order to accommodate mineralization—a process termed matrix maturation. This maturation phase includes cleavage of C- and N-terminal propeptides from the collagen molecule and collagen crosslinking and packaging (Knott and Bailey, 1998; Christiansen et al., 2000), as well as similar processing of noncollagenous proteins (Kaartinen et al., 2002). The process of extracellular matrix maturation lasts 10–14 days. For this model, we considered different steps of matrix maturation such as post-translational modification of collagen and noncollagenous matrix proteins, and collagen cross-linking as a maturation process, which normally occurs with a characteristic rate constant of *k*_1_. In the model, a change in any step of post-translational modification or crosslinking is assumed to have an overall effect on matrix maturation by either facilitating or interfering with the normal process. We assumed that collagen matrix is produced by osteoblasts in a naïve form (*x*_1_) that matures into a fully assembled mature collagen matrix (*x*_2_). These relationships are described by Equations (1a) and (1b).
2. The mineralization of naïve matrix is prevented by the action of numerous inhibitors, which reside in the local bone extracellular microenvironment, or arrive from the circulation (Murshed and McKee, 2010). During matrix maturation, the inhibitors are degraded or inactivated to enable mineralization. For example, a potent small-molecule inhibitor of mineralization—inorganic pyrophosphate—is cleaved by alkaline phosphatase present on the osteoblast cell membrane (Murshed and McKee, 2010). The proteins of the SIBLING family (small integrin-binding ligand, N-linked glycoproteins) are also known for their role in the regulation of mineralization; for example, the SIBLING proteins osteopontin (OPN) and matrix extracellular phosphoglycoprotein (MEPE) are potent inhibitors of mineralization (Rowe et al., 2004; Jahnen-Dechent et al., 2008). The action of another SIBLING protein dentin matrix protein 1 (DMP1) is regulated by its state during matrix maturation. DMP1 acts an inhibitor of mineralization when it is in solution (He et al., 2005), but becomes a promoter of mineralization when absorbed onto collagen surfaces (Hunter and Goldberg, 1993; Hunter et al., 1996; He et al., 2003). We modeled the combined action of different inhibitors of mineralization (*I*), which were assumed to be released into the extracellular compartment near the cells (Murshed and McKee, 2010) and diffuse through immature collagen (Weinstock and Leblond, 1973) with the characteristic rate constant of *v*_1_. Thus, inhibitor availability was modeled to be proportional to the amount of naïve collagen as described by the term *v*_1_*x*_1_ in the Equation (1c). The concentration of active inhibitors in mineralizing matrix gradually decreases both because of their degradation through enzymatic cleavage (Addison et al., 2008, 2010; Murshed and McKee, 2010; Barros et al., 2013) as well as removal by other processes that interfere with inhibitor function, such as binding, masking or trapping (He et al., 2005; David et al., 2011). In the model, inhibitor removal/reduction occurs with the rate constant of *r*_1_ and is stimulated by the presence of mature collagen matrix and as described by the term *r*_1_*x*_2_*I* in the Equation (1c).
3. During the mineralization process calcium and phosphate precipitate to form hydroxyapatite [Ca_10_(PO_4_)_6_(OH)_2_] crystals within the organic bone matrix (Boskey and Posner, 1984). The location and orientation of individual crystals is not random, but rather is guided by the chemistry and structure of collagen and noncollagenous proteins and small proteoglycans initiating and regulating crystal nucleation and growth between and within collagen fibrils (George and Veis, 2008). Within the collagen fibril the mineral is formed in-between the assembled collagen molecules (intrafibrillar mineralization) (George and Veis, 2008). Interfibrillar crystals can be nucleated by the SIBLING proteins bone sialoprotein and DMP1 (Hunter and Goldberg, 1993; Hunter et al., 1996; He et al., 2003). We assumed that nucleation centers (*N*) are required to initiate mineral precipitation (Hunter and Goldberg, 1993; He et al., 2003), and that nucleators appear during matrix maturation. The number of nucleators per mature collagen molecule is *k*_2_. We assumed that intrafibrillar and interfibrillar nucleators act in a similar way, therefore when *k*_2_ = 1, there is only one intrafibrillar nucleator per 1 molecule of collagen, and when *k*_2_ > 1, then there is a mix of intrafibrillar and interfibrillar nucleators. A resulting rate of nucleator appearance is proportional to matrix maturation given by Equation (1b) and is described by the term $k_{2}\frac{dx_{2}}{dt}$ in Equation (1d). We assume that after mineralization is initiated by a given nucleator, this nucleator becomes a mineral crystal and thus can maintain, but no longer can initiate mineral precipitation (Hunter et al., 1996). Therefore, when mineraization starts, the number of nucleators decreases as they become masked by the mineral. The rate of decrease of nucleators was assumed to be proportional to the rate at which mineralized crystals (*y*) appear (*dy/dt*), as well as to the concentration of nucleators present, as described by the term $r_{2}\frac{dy}{dt}N$ in Equation (1d).
4. The formation of mineral (*y*) was assumed to occur with a characteristic rate of *k*_3_ and to be directly proportional to the number of nucleators and inversely related to the amount of inhibitors (Murshed and McKee, 2010). Although we modeled matrix mineralization in a homogenous assumption, it would be possible to relate the number of nucleators *N* to the number of mineral crystals within a given volume of the matrix, while the mineral growth rate *k*_3_ to the growth of individual crystals. Particular considerations were given to the function describing the effect of inhibitors on mineral formation. We modeled mineralization rate by an equation of the form $\frac{dy}{dt}=k_{3}g\left(I\right)N$, where *g(I)* is a decreasing function of *I* which tends to 0 as *I* goes to infinity. Mineralization dynamics was qualitatively similar when *g(I)* was described by the piecewise function $g(I)=\{\begin{array}{c} -aI+b,x\leq 1\\ 0,I>1 \end{array}\}$ or the Hill type functions $g\left(I\right)=\frac{b}{b+e^{aI}}$ and $g\left(I\right)=\frac{b}{b+I^{a}}$, (data not shown). We chose a differentiable function amenable for biological interpretation $g\left(I\right)=\frac{b}{b+I^{a}}$ with *a* = 10 and *b* = 0.001. This function approaches 1 at *I* smaller than ~0.4, which represents the critical (nondimensionalized) value of *I* permitting mineralization in the system.

**Figure 1:**
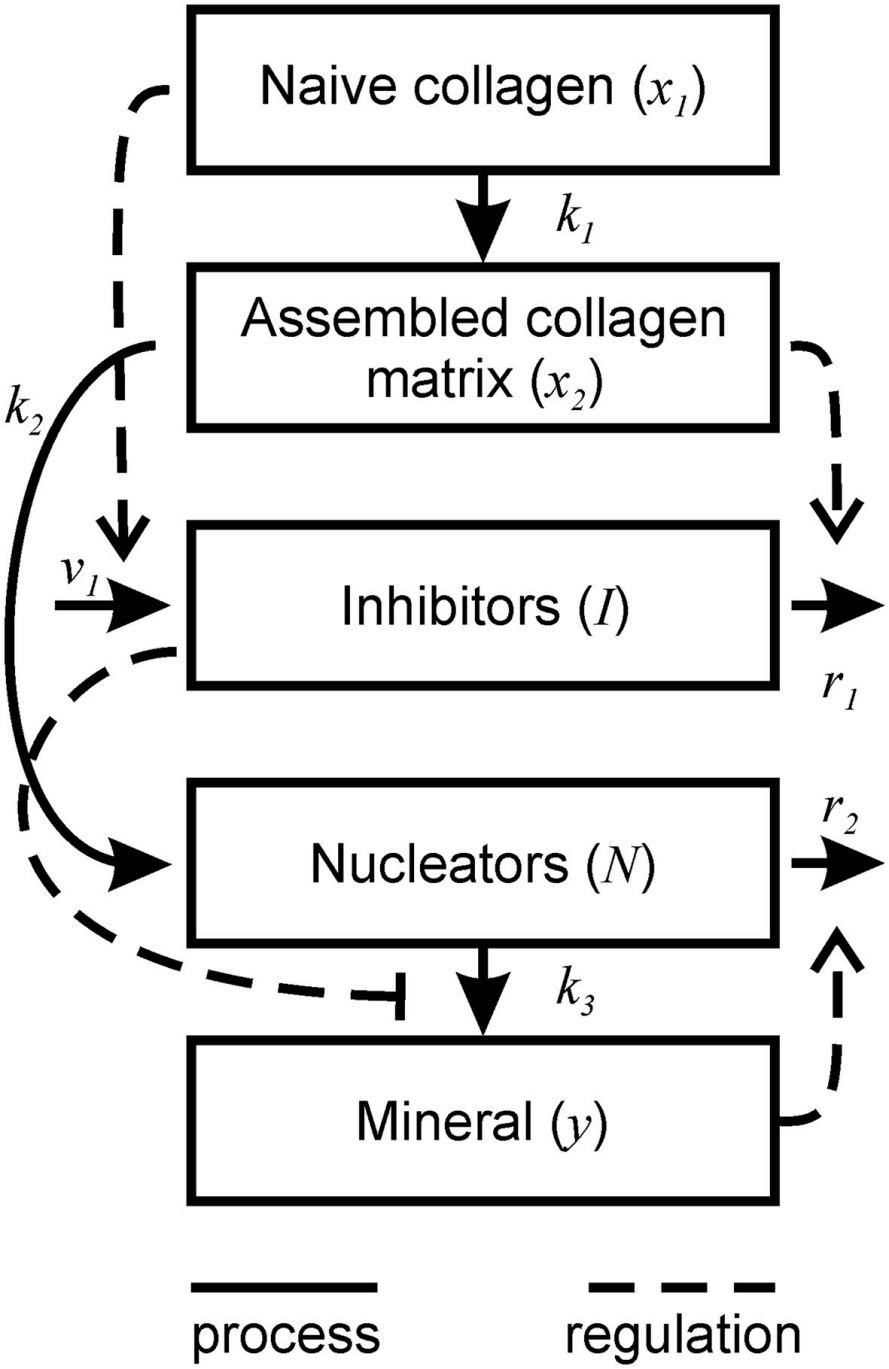
**Schematic representation of bone mineralization described by the model**. Thick lines represent the processes occurring during mineralization. Dotted lines represent the regulatory effects of different components on the mineralization process.

Based on these assumptions (Figure 1), the changes in the five components of the mineralizing bone matrix (Table 1) are described by the following system of ordinary differential Equations (1).

(1a) $$\begin{array}{l} \frac{dx_{1}}{dt}=-k_{1}x_{1} \end{array}$$

(1b) $$\begin{array}{l} \frac{dx_{2}}{dt}=k_{1}x_{1} \end{array}$$

(1c) $$\begin{array}{l} \frac{dI}{dt}=v_{1}x_{1}-r_{1}x_{2}I \end{array}$$

(1d) $$\begin{array}{l} \frac{dN}{dt}=k_{2}\frac{dx_{2}}{dt}-r_{2}\frac{dy}{dt}N \end{array}$$

(1e) $$\begin{array}{l} \frac{dy}{dt}=k_{3}\left(\frac{b}{b+I^{a}}\right)N \end{array}$$

**Table 1:** **Variables used in Equation (1)**.

**Variables**	**Concentration represented**	**Characteristic values**
*x*_1_	Collagen matrix (molecules/μm^3^)	9.4 × 10^5^ molecules/μm^3^
*x*_2_	Assembled collagen matrix (molecules/μm^3^)	9.4 × 10^5^ molecules/μm^3^
*I*	Inhibitor concentration (molecules/μm^3^)	~10^6^ molecules/μm^3^
*N*	Nucleator concentration (molecules/μm^3^)	1–10 per 1 assembled collagen
*y*	Hydroxapatite (molecules/μm^3^)	0.8 × 10^9^ molecules/μm^3^

### Estimation of characteristic values of the variables and parameters

To estimate collagen packing within 1 μm^3^ of matrix, we assume that a single molecule of triple-helical collagen is 1.4 nm in diameter and 300 nm in length based on estimates in the literature (Gross et al., 1955; George and Veis, 2008). Collagen molecules form fibrils of 70–90 nm in diameters, thus a single fibril contains ~3000 molecules (Hodge and Schmitt, 1960; George and Veis, 2008). We assume that fibrils have a ~10 nm coating of noncollagenous proteins and small proteoglycans, thus the cross-section of collagen fibrils is represented by circles of 110 nm diameter. In a hexagonal pattern circles have ~0.9069 packing density (Steinhaus, 1999), and hence, (0.9069 × 10^6^/(π × 55^2^) ~ 95.4 fibrils fit in a cross-section of 1 μm^2^, and ~3.3 molecules fit in the 1 μm lengthwise. Therefore, in 1 μm^3^ of volume, there are 95.4 × 3.3 × 3000 = 9.4 × 10^5^ molecules of collagen.

To estimate the number of hydroxyapatite molecules, we started with a density of fully mineralized bone of 2.0 g/cm^3^ (Gong et al., 1964). Assuming that bone contains 70% mineral, the hydroxyapatite density is ~1.40 g/cm^3^ = 1.40 × 10^−12^ g/μm^3^. Given the molecular weight of the hydroxyapatite molecule [Ca_10_(PO_4_)_6_(OH)_2_] of 1004 g/mol, 1004 g contains 6 × 10^23^ molecules of hydroxyapatite. Therefore, 1 μm^3^ of mineralized matrix contains ~ 0.8 × 10^9^ molecules of hydroxyapatite.

The number of nucleators was first assumed to be of the same order of magnitude as the number of collagen molecules (*k*_2_ = 1), and later we examined how changing the number of nucleators affects the outcome of the mineralization. It is difficult to estimate the number of inhibitors, since we pooled into this category factors that use different mechanisms to achieve a single function—inhibition of mineralization. These factors are much smaller in size than the collagen molecules; however, there is less physical space available for them, and therefore we assumed that the numbers of inhibitor molecules and collagen molecules are of the same order of magnitude.

The rate constant values were chosen based on the observation that two main phases are present during bone mineralization: a slow phase of matrix maturation and a relatively fast phase of matrix mineralization (Boskey and Posner, 1984; George and Veis, 2008; Murshed and McKee, 2010). To account for this dynamic, we assumed that the rates of matrix maturation and the related processes of inhibitor processing and nucleator production are slower than the rates of mineral precipitation and nucleator removal/reduction. Since collagen maturation takes place by ~10–14 days (Boskey and Posner, 1984; George and Veis, 2008), the rate of collagen assembly *k*_1_ was estimated as 0.1 day^−1^. We assumed the rate of inhibitor delivery to be *v*_1_ = 0.1 day^−1^ and the rate of inhibitor degradation to be *r*_1_ = 2 × 10^−7^ day^−1^ mol^−1^. We assumed that when nucleators are available, and no inhibitors are present, mineralization occurs with a faster rate than collagen assembly *k*_3_ = 1000 day^−1^. An order of magnitude for the rate of the nucleator use by mineralization was *r*_2_ = 1.5–2 × 10^−8^ mol^−1^. The parameter values for the simulation of normal mineralization are given in Table 2. Further details for model nondimensionalization and numerical analysis are given in Supplementary Material.

**Table 2:** **Parameters used in Equations (1) and (2)**.

**Parameter**	**Description**	**Value**	**Nondimensionalized**
*k*_1_	Collagen assembly	0.1 day^−1^	0.1
*k*_2_	Number of nucleators per collagen molecule	1	1
*k*_3_	Formation of hydroxyapatite molecules	1000 day^−1^	1
υ_1_	Production of inhibitors by osteoblasts	0.1 day^−1^	0.1
*r*_1_	Degradation of inhibitors	2 × 10^−7^ day^−1^	0.2
*R*_2_	Use of nucleators by mineralized bone	1.7 × 10^−8^mol^−1^	12
*a*	Hill coefficient	10	10
*b*	Apparent dissociation constant for Hill function	10^57^	0.001

## Results

**Figure 2:**
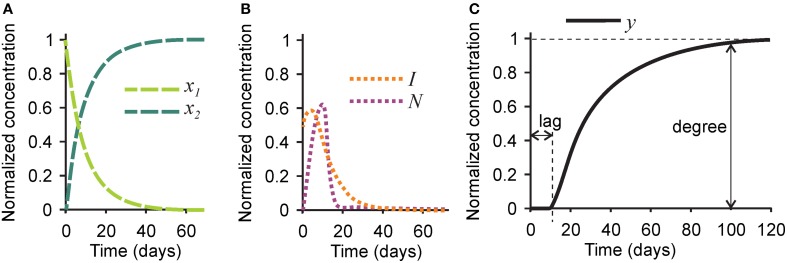
**Changes in time in different players in the mineralization process in healthy bone**. **(A)** The concentrations of naïve (*x*_1_, light green) and mature (*x*_2_, dark green) collagen matrix. **(B)** The concentration of the mineralization inhibitor (*I*, orange) and the nucleation centers (*N*, purple). **(C)** The concentration of mineral (*y*). Indicated are the mineralization lag time, measured as a time delay between time 0 and the onset of mineralization, and mineralization degree, measured as the amount of mineral at time = 100 days. For the simulation of healthy bone, the mineralization lag time is 10 days, and the mineralization degree is 1.

First, we examined the pattern of temporal changes in the five variables for the parameters representing bone mineralization in a healthy subject (Tables 1, 2). Naïve collagen, which initially constituted 100% of all collagen in the system, was gradually assembled into mature collagen, resulting in 80% conversion within 20 days, and in complete maturation within 40–60 days (Figure 2A). Inhibitors initially present in the naïve matrix were sustained for the first 10 days and rapidly degraded with the appearance of mature collagen (Figure 2B). Nucleating centers produced with the mature collagen reached the maximum at ~10 days, and were removed with the offset of the mineralization (Figure 2B). After a lag time of ~10 days, the mineralization first progressed rapidly followed by a continuous slow mineral formation (Figure 2C). The normalized mineralization degree of 1 (i.e., full mineralization) was reached ~100 days after the deposition of naïve collagen. Thus, the model describes the lag time required for matrix maturation, the rapid mineralization offset, and the continuous slow increase in mineralization with time (Roschger et al., 2008b).

**Figure 3:**
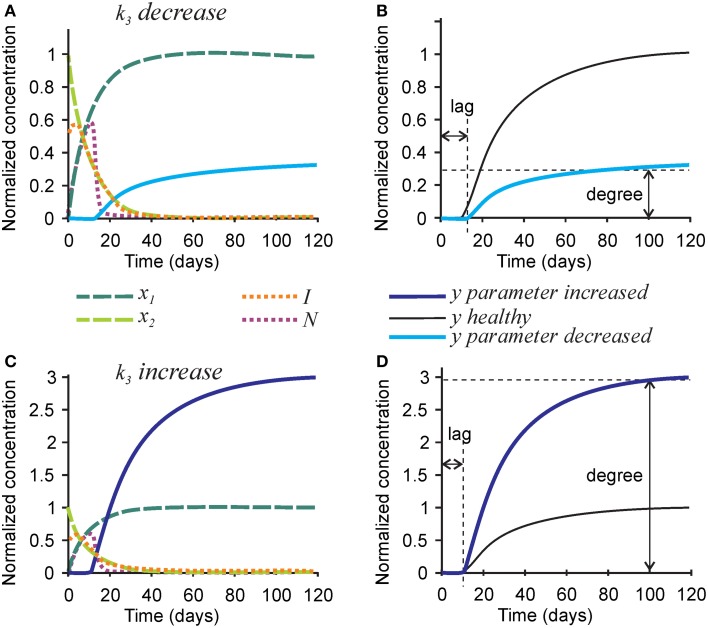
**The effect of parameter affecting formation of hydroxyapatite crystals *k*_3_ on the mineralization outcome**. **(A)** The effect of decreasing *k*_3_ 3-fold. **(B)** Comparison of the mineralization lag time and degree following decrease in *k*_3_ to healthy mineralization. **(C)** The effect of increasing *k*_3_ 3-fold. **(D)** Comparison of the mineralization lag time and degree following increase in *k*_3_ to healthy mineralization. The same color scheme is used as in Figure 2.

To model mineralization defects, we first examined the effect of the rate of hydroxyapatite formation *k*_3_ on the mineralization outcome (Figure 3). Changes in *k*_3_ predictably affected the rate of mineral formation, but also strongly and proportionally affected the degree of mineralization. A 3-fold decrease in the rate of hydroxyapatite formation *k*_3_ resulted in a 3-fold decrease in mineralization degree (Figures 3A,B), while a 3-fold increase in *k*_3_ led to a 3-fold increase in mineralization degree (Figures 3C,D). The robust effect of *k*_3_ on the degree of mineralization is due to the fact that the removal of nucleators from the model is regulated by two independent parameters—the rate of hydroxyapatite formation (directly affected by *k*_3_), and the efficiency of nucleator removal (*r*_2_), which remains high even when *k*_3_ is low. Therefore, when the rate of hydroxyapatite formation decreases, the time interval during which nucleators are present remains unchanged, resulting in a decrease in mineralization degree. Changes in *k*_3_ did not affect the dynamics of collagen maturation or turnover of its inhibitors.

**Figure 4:**
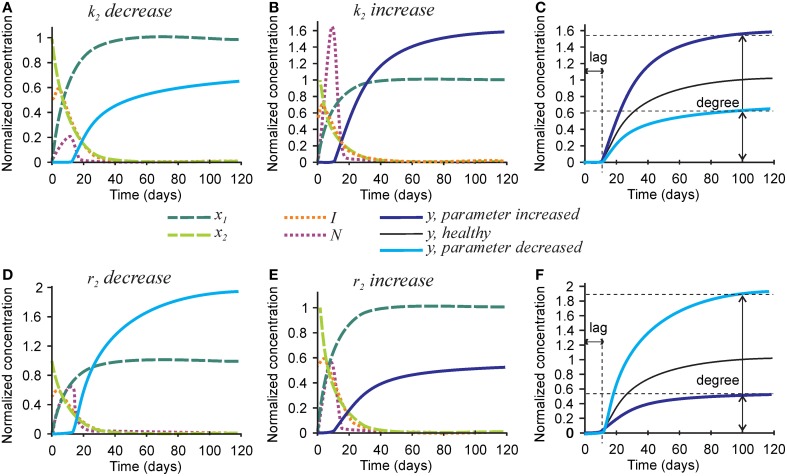
**The effect of parameters affecting nucleator production and removal on the mineralization outcome**. **(A–C)** The effect of decreasing 3-fold **(A)** or increasing 3-fold **(B)** the number of nucleators per crosslinked collagen (*k*_2_). **(C)** Comparison of the mineralization lag time and degree in conditions affecting *k*_2_ to healthy mineralization. **(D–F)** The effect of decreasing 3-fold **(D)** or increasing 3-fold **(E)** the rate of use of nucleators by mineralized bone (*r*_2_). **(F)** Comparison of the mineralization lag time and degree in conditions affecting *r*_2_ to healthy mineralization. The same color scheme is used as in Figure 2.

Next we examined the role of parameters affecting the nucleators, the number of nucleators per mature collagen *k*_2_, and rate of removal of nucleators caused by hydroxyapatite formation *r*_2_ (Figure 4). Mineralization lag time was not affected by changes in *k*_2_ and *r*_2_, since the dynamics of collagen and inhibitors does not depend on these parameters (Figures 4C,F). A 3-fold decrease in the number of nucleators per mature collagen *k*_2_ resulted in a 40% decrease in mineralization degree (Figures 4A,C), while a 3-fold increase in *k*_2_ led to a 60% increase in the mineralization degree (Figures 4B,C). A 3-fold decrease in the rate of nucleator removal caused by hydroxyapatite formation *r*_2_ resulted in an almost 2-fold increase in mineralization degree (Figures 4D,F), while a 3-fold increase in *r*_2_ resulted in 40% decrease in mineralization degree (Figures 4E,F).

**Figure 5:**
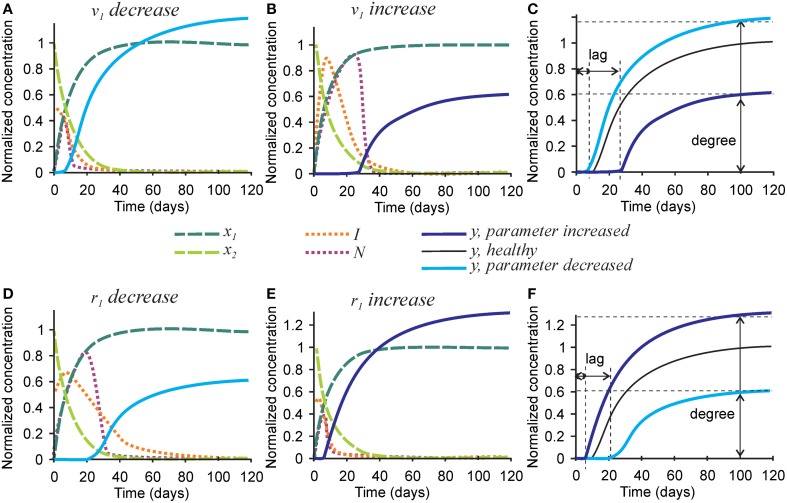
**The effect of parameters affecting inhibitor production and degradation on the mineralization outcome**. **(A–C)** The effect of decreasing 10-fold **(A)** or increasing 10-fold **(B)** the rate of inhibitor production (*v*_1_). **(C)** Comparison of the mineralization lag time and degree in conditions affecting *v*_1_to healthy mineralization. **(D–F)** The effect of decreasing 3-fold **(D)** or increasing 3-fold **(E)** the rate of inhibitor degradation (*r*_1_). **(F)** Comparison of the mineralization lag and degree in conditions affecting *r*_1_ to healthy mineralization. The same color scheme is used as in Figure 2.

To examine the effect of parameters affecting the homeostasis of inhibitors on the mineralization outcome, we changed the rates of inhibitor production *v*_1_ and degradation *r*_1_ (Figure 5). Since Equations (1a) and (1b) are not affected by these parameters, no change in the degree or timing of collagen maturation was evident following changes in *v*_1_ and *r*_1_. The rate of inhibitor production *v*_1_ was changed 10-fold since smaller changes only resulted in slight differences in the mineralization. A 10-fold decrease in the rate of inhibitor production *v*_1_ resulted in a ~20% decrease in mineralization lag time and a similar 20% increase in mineralization degree (Figures 5A,C). A 10-fold increase in the rate of inhibitor production *v*_1_ led to a 3-fold increase in mineralization lag time and a 40% decrease in mineralization degree (Figures 5B,C). The effect of changing the rate of inhibitor degradation *r*_1_ on mineralization mirrored the effects of changing the rate of inhibitor production *v*_1_, however, smaller, 3-fold alterations of *r*_1_ were required to obtain noticeable effects on mineralization. A 3-fold decrease in *r*_1_ resulted in a sustained inhibitor presence, a 2-fold increase in mineralization lag time and 40% decrease in mineralization degree (Figures 5D,F). A 3-fold increase in the rate of inhibitor degradation *r*_1_ resulted in a 2-fold decrease in the mineralization lag time and 20% increase in mineralization degree (Figures 5E,F).

**Figure 6:**
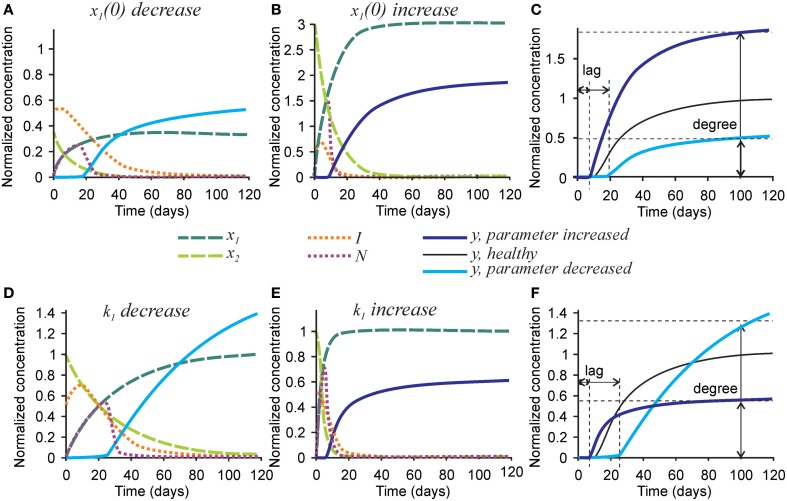
**The effect of parameters affecting collagen maturation on the mineralization outcome**. **(A–C)** The effect of decreasing 3-fold **(A)** or increasing 3-fold **(B)** the amount of naïve collagen deposited by osteoblasts at time = 0 *(x_1_(0))*. **(C)** Comparison of the mineralization lag time and degree in conditions affecting *x_1_(0)* to healthy mineralization. **(D–F)** The effect of decreasing 3-fold **(D)** or increasing 3-fold **(E)** the rate of collagen maturation (*k*_1_*)*. **(F)** Comparison of the mineralization lag and degree in conditions affecting *k*_1_ to healthy mineralization. The same color scheme is used as in Figure 2.

Finally, we examined the effect of changing the parameters affecting initial collagen density *x*_1_(0) and maturation *k*_1_ on the mineralization outcome (Figure 6). Change in the initial density of naïve collagen *x*_1_(0) represents an altered ability of osteoblasts to produce collagen, or altered collagen packing. A 3-fold decrease in *x*_1_(0) resulted in a proportionally lower amount of mature collagen and the number of nucleators, leading to a 2-fold decrease in mineralization degree (Figures 6A,C). In addition, the inhibitor presence was sustained for a longer period of time leading to a 2-fold increase in mineralization lag time (Figure 6A). A 3-fold increase in *x*_1_(0) led to a 3-fold increase in the amount of mature collagen and in the number of nucleators, which however translated to only a 70-80% increase in mineralization degree (Figures 6B,C).

A 3-fold decrease in the rate of collagen maturation *k*_1_ resulted in the persistence of naïve collagen for up to 100 days and sustained inhibitor presence, leading to an almost 3-fold increase in mineralization lag time (Figure 6D). After mineralization started, it proceeded slower in the initial phase than in control conditions (Figures 6D,F). However, slow delivery of nucleators into the system resulted in a decrease in the rate of their removal (when nucleators are present at a low density, each of them can participate in mineralization for a longer time since they interfere less with each other). As a result, the mineralization rate did not decrease with time and a notably increased mineralization degree was reached (Figures 6D,F). A 3-fold increase in the rate of collagen maturation resulted in faster elimination of inhibitors and a slightly decreased mineralization lag time. The initial mineralization proceeded faster; however, because of faster removal of nucleators, it leveled off at lower overall mineralization degree (Figures 6E,F).

## Discussion

The mathematical model for bone mineralization developed in this study captures the strongly nonlinear dynamics of mineralization, which starts from a lag phase when osteoid is present but no mineralization is evident, followed by fast primary mineralization, and subsequent secondary mineralization characterized by a continuous slow increase in bone mineral content (Roschger et al., 2008b). This dynamic was achieved in the model by assuming that (*i*) mineralization is suppressed in the presence of inhibitors, (*ii*) mineralization occurs fast, but requires the presence of nucleators, and (*iii*) nucleators formed during collagen maturation are removed from the system proportionally to the rate of mineralization. As a result, the lag phase allows for accumulation of nucleators, so that when inhibitors are reduced a large number of nucleators are present allowing mineralization to proceed rapidly. However, fast mineralization causes fast removal of nucleators leading to a substantial decrease in mineralization rate with time. We examined how changes in different parameters affect mineralization dynamics. The parameters describing the formation of hydroxyapatite crystals at the nucleating centers potently affected the degree of mineralization, while the parameters describing inhibitor homeostasis effectively changed the mineralization lag time. Of interest, a single parameter describing the rate of matrix maturation was capable of counter-intuitively increasing both the mineralization lag time and the degree of mineralization.

The model represents an intentional simplification of a complex mineralization process, as we focused on simultaneously capturing mineralization-related functions of many regulatory molecules. Therefore, the following limitations should be noted: (1) The model does not specify different steps of matrix maturation, such as post-translational modification of collagen and noncollagenous matrix proteins, and collagen crosslinking. (2) The action of a large number of chemically distinct inhibitors is pooled together as a single entity. (3) Similarly, the difference in action of intrafibrillar and interfibrillar nucleators is not described. (4) The model does not contain the physical limitation for the maximal amount of mineral that can be deposited into the matrix, and therefore its long-term predictions should be interpreted with caution. Model applicability at this stage is limited to situations when the changes in mineralization dynamics are dramatic, while further development of the model is required to predict more subtle changes over time such as occurring during development and in complex disorders of osteoporosis and diabetes.

To compare the model predictions to the phenotype of bone disorders known to result in abnormal mineralization, we examined hypomineralization in osteomalacia and hypermineralization in osteogenesis imperfecta. We used the proportion of osteoid (osteoid volume per bone volume OV/BV, and/or osteoid thickness O.Th.) as an indicator of mineralization lag time, and bone mineral density distribution (BMDD) (Roschger et al., 2008b) as a measure of mineralization degree to relate the disease mineralization phenotype observed on histomorphometric and BMDD analysis to model predictions.

### Application of the model to osteomalacia

Osteomalacia arises in part because of a systemic deficiency in calcium and/or phosphate ions and the hormones responsible for their regulation—vitamin D and FGF23. It is characterized by an increase in mineralization lag time and a decrease in mineralization degree (Arnala et al., 2001; Roschger et al., 2003; Rabelink et al., 2011; Cheung et al., 2013). It is assumed that the main cause of osteomalacia is a decreased rate of hydroxyapatite formation (reflected by the parameter *k*_3_ in the model) caused by a low level of calcium and/or phosphate. However, the model predicts that a decrease in *k*_3_ accounts only for a strong decrease in mineralization degree, but cannot by itself affect the mineralization lag time (as mineral formation starts only after the lag phase is completed). In order to account for the strong increase in the mineralization lag time, it is necessary to assume additional direct or indirect effects of calcium/phosphate deficiency on inhibitor homeostasis. In fact, increases in local extracellular matrix mineralization inhibitors were also shown to contribute to the development of osteomalacia (Harmey et al., 2006; Barros et al., 2013; Millán, 2013). Inorganic calcium and phosphate are known to affect osteoblast differentiation (Beck et al., 2003; Dvorak et al., 2004), which could in turn result in changes in expression and processing of mineralization inhibitors. Deficiency in active 1,25-dihydroxyvitamin D (1,25(OH)2D) often associated with rickets (Takeda et al., 1997; Fukumoto, 2014) can affect the vitamin D receptor-mediated expression of mineralization inhibitors such as DMP1 (Nociti et al., 2014). Moreover, degradation of a strong inhibitor of mineralization—pyrophosphate (Addison et al., 2007)—is regulated by the concentration of phosphate, and both phosphate and pyrophosphate regulate expression of osteopontin (Harmey et al., 2006; Addison et al., 2007). In this context, removal/reduction of inhibitory osteopontin and its inhibitory peptides can be achieved by their extensive degradation by the enzyme PHEX (Addison et al., 2010; Barros et al., 2013). The model suggests that alteration of local inhibitor homeostasis is as important for the development of osteomalacia as is the direct effect of low calcium and phosphate on the rate of hydroxyapatite formation.

### Application of the model to osteogenesis imperfecta

Osteogenesis imperfecta (OI) is a disease characterized by high bone fragility associated with low bone mass as well as high mineral content in the bone tissue resulting in its brittleness (Roschger et al., 2008a). Mutations in genes coding for collagen type I—the usual cause of osteogenesis imperfecta—are associated with hypermineralization and normal mineralization lag time (Rauch et al., 2000). In the model, an increase in the number of nucleators per molecule of collagen (*k*_2_) results in an increase in mineralization degree but does not affect the mineralization lag time. Therefore, the model suggests that the hypermineralization in OI caused by mutations in type I collagen-encoding genes is attributable to the increase in the number of nucleators per molecule of collagen. This prediction is consistent with a recent study that demonstrated that the hydroxyapatite crystal size is similar in OI and control bone tissue, thus implying that the increased mineral content in OI must be due to an increased density of mineral crystals (Fratzl-Zelman et al., 2014). Since in the model the density of nucleating centers corresponds to the density of mineral crystals, we conclude that the model correctly predicts hypermineralization in OI due to mutations in genes coding for collagen type I.

Of interest, there are two distinct forms of OI in which different mineralization phenotypes are described. In OI caused by mutations in cartilage-associated protein (CRTAP) a significant increase in the mineralization degree (Fratzl-Zelman et al., 2010) and a marked reduction in mineralization lag time (Morello et al., 2006) were observed. In contrast, mutations in the collagen type I C-propeptide cleavage site give rise to a hypermineralization accompanied by a simultaneous increase in the mineralization lag time (Lindahl et al., 2011).

CRTAP forms a complex with P3H1 and cyclophilin B which 3-hydroxylates the Pro986 residue of collagen alpha chains (Chang et al., 2010). It was reported that CRTAP deficiency results the deposition of abnormally structured collagen fibrils (variable in diameter, with irregular borders) in skin samples of OI patients due to CRTAP mutation (Valli et al., 2012). The model predicts that an increase in the initial collagen density (*x*_1_(0)) can result in hypermineralization accompanied by a significant decrease in mineralization lag time. It is important to stress that the model describes the changes occurring in the already-deposited collagen, but not the rate of its deposition by osteoblasts, which is negatively affected by CTRAP mutation. It is indeed noticeable, that the distance between the collagen fibers in the skin of a patient with CRTAP mutation appear to be smaller (Valli et al., 2012). Thus, the CRTAP mutation likely affects the packing of collagen molecules simultaneously resulting in (*i*) an increase in trapping/masking and degradation of inhibitors, thus shortening the mineralization lag time, and (*ii*) an increase in the density of nucleators leading to an increase in mineralization degree.

Mutation in the collagen C-propeptide cleavage site disrupts extracellular collagen processing, resulting in decreased collagen maturation rate (Lindahl et al., 2011), represented in our model by the parameter *k*_1_. In the model, decrease in *k*_1_ uniquely gave rise to the phenotype of increase in both mineralization lag time and degree. Conversely, mutation in *BMP1*—an enzyme that cleaves C-propeptide off procollagen—also results in a decrease in collagen maturation, hyperosteoidosis and hypermineralization (Hoyer-Kuhn et al., 2013). Thus, our model predicted a correct, albeit counter-intuitive, mineralization phenotype resulting from a decrease in the collagen matrix maturation rate.

## Conclusion

We have developed a simplified mathematical model that describes changes in the mineralization of bone matrix when individual processes occurring during mineralization are altered. We validated the accuracy of model predictions using bone diseases associated with dramatic changes in mineralization dynamics. During model development we used the data relevant to the mineralization process in human bone and applied the model to the analysis of human disorders of bone mineralization. In the future, this model can be applied for qualitative predictions of genotype/phenotype relationship in mouse models of bone mineralization, and it can be adapted to study mineralization of other calcified tissues, such as tooth dentin, cementum and enamel.

## Author contributions

SK, MDM, MM, and FR developed a biological framework of the model; LS, JG, EZ, MO, and SK constructed the model; LS, JG, EZ analyzed the model and generated the figures; SK prepared the first draft, all the co-authors read, critically revised, and approved the final manuscript.

### Conflict of interest statement

The authors declare that the research was conducted in the absence of any commercial or financial relationships that could be construed as a potential conflict of interest.
