# Supplementary material for: Mathematical model for bone mineralization
Source: Front Cell Dev Biol. 2015 Aug 21;3:51. doi: 10.3389/fcell.2015.00051 (PMC4544393; doi:10.3389/fcell.2015.00051)
Supplement: Supplementary file 1 [file DataSheet1.PDF]

Supplementary material for the manuscript "Mathematical model for bone mineralization" by SV Komarova, L Safranek, J Gopalakrishnan, M Y Ou, MD McKee, M Murshed, F Rauch, E Zuhr.

Nondimensionalization

We scaled the variables representing concentrations of collagen matrix state variables, the inhibitors, and the nucleators by the constant characteristic values of $\hat{x} = 10^6$ molecules/μm^3 and we scaled the concentration of the hydroxyapatite by $\hat{y} = 10^9$ molecules/μm^3. The system which arises after the substitutions $x^*_{1,2} = \frac{x_{1,2}}{\hat{x}}$; $I^* = \frac{I}{\hat{x}}$; $N^* = \frac{N}{\hat{x}}$; $y^* = \frac{y}{\hat{y}}$ is given in equation (2), where $\hat{r_1} = r_1\hat{x}$; $\hat{r_2} = r_2\hat{x}$; $\hat{b} = \frac{b}{\hat{x}^a}$.

$$\frac{dx_1^*}{dt} = -k_1 x_1^* \qquad (2a)$$

$$\frac{dx_2^*}{dt} = k_1 x_1^* \qquad (2b)$$

$$\frac{dI^*}{dt} = v_1 x_1^* - \hat{r_1} x_2^* I^* \qquad (2c)$$

$$\frac{dN^*}{dt} = k_1 \frac{dx_2^*}{dt} - \hat{r_2} \frac{dy^*}{dt} N^* \qquad (2d)$$

$$\frac{dy^*}{dt} = k_3 \left(\frac{\hat{b}}{\hat{b} + I^{*a}}\right) N^* \qquad (2e)$$

Nondimensionalization in time was also calculated, but this simplification of the system came at the cost of obscuring the role of the parameter k_1, which will be shown in the results section to be one of the important parameters of the system.

Numerical simulations

Numerical simulations were performed using the built in MATLAB solver for stiff ordinary differential equations, ode15s, to solve the nondimensionalized system given in Equation (2). Initial conditions were $x_1(0) = 1$; $x_2(0) = 0$; $I(0) = 0.5$; $N(0) = 0$; $y(0) = 0$ unless noted otherwise. Parameter values for normal bone mineralization are from the computational column of Table 2.

Matlab code for simulating and plotting solutions

```
function pedfpub()
% Program to solve system of ordinary differential equations
% modeling bone mineralization

  % Nondimensionalized parameter values:
  % Fixed Values
  k1 = 0.1; % Inversely related to time lag and max mineralization
            % (Decreasing k1 by a factor of 3 increases time lag
            % to 40 days.)
  k2 = 1;   % This value is fixed.
  k3 = 1;   % Directly related to max mineralization.
  v1 = 0.1;  % Directly affects max I, and inversely affects time-
             % lag in some range of values of r1.

  % Values fit to healthy model
  r1 = 0.2;  % Affects time lag (inversely) & mineralization
             % (directly)
  r2 = 17;  % Affects mineralization (inversely)
  a  = 10;
  b  = 0.001; %



  % Calculation of physical parameter values from
  % nomdimensionalized parameter values
  xhat = 1e6;
  yhat = 1e9;
  k1_physical = k1;
  v1_physical = v1;
  r1_physical = r1/xhat;
  k2_physical = k2;
  k3_physical = k3*yhat/xhat;
  r2_physical = r2/(k3_physical*xhat);


    fprintf('PARAMETERS:  Nondimensional     Physical ¥n');
  fprintf('--------------------------------------------¥n');
  fprintf('    k1:      %13e    %13e¥n',k1,k1_physical);
  fprintf('    k2:      %13e    %13e¥n',k2,k2_physical);
  fprintf('    k3:      %13e    %13e¥n',k3,k3_physical);
  fprintf('    r1:      %13e    %13e¥n',r1,r1_physical);
  fprintf('    r2:      %13e    %13e¥n',r2,r2_physical);
  fprintf('    v1:      %13e    %13e¥n',v1,v1_physical);


% Nondimensionalized equations
    function Zdot = rhs(t,z) %z=(x1,x2,I,N,y)
        Zdot=zeros(5,1);
```

```
        Zdot(1) = -k1*z(1);
        Zdot(2) = k1*z(1);
        Zdot(3) = v1*z(1) - r1*z(2)*z(3);
        Zdot(4) = k1*k2*z(1) - r2*H(z(3))*z(4)*z(4);
        Zdot(5) = k3*H(z(3))*z(4);
    end


    % Modifed Hill function
    function hill = H(x)
        hill = b/(b+x^a);
    end

 % Implement built in ODE solver
 %                      t0, t1    X1,X2,I, N, Y
  [T,Y] = ode15s(@rhs, [0, 120], [1, 0,1, 0, 0]);

  % Plot results
  Imax = ceil (max( Y(:,3) ) );
  plot(T,Y(:,1),'c:',      'LineWidth',2) ;hold on
  plot(T,Y(:,2),'g-.',     'LineWidth',2) ;hold on
  plot(T,Y(:,3)/Imax,'r:','LineWidth',2) ;hold on
  plot(T,Y(:,4),'m--',     'LineWidth',2) ;hold on
  plot(T,Y(:,5),'b-',      'LineWidth',2) ;hold on
  strI = strcat('I/',sprintf('%d: Inhibitor/Max',Imax));
  legend('X_1: Collagen','X_2: Crosslinked',strI,...
         'N: Nucleator','Y: Mineral', ...
         'Location', 'SouthEast');
  axis( [0,120,0,1.2] )
  xlabel('Time in days')
  ylabel('Concentration')
end
```
